# Vaccination in England: a review of why business as usual is not enough to maintain coverage

**DOI:** 10.1186/s12889-018-6228-5

**Published:** 2018-12-06

**Authors:** Tim Crocker-Buque, Sandra Mounier-Jack

**Affiliations:** Faculty of Public Health and Policy, London School of Hygiene and Tropical Medicine, 15-17 Tavistock Place, London, WC1H9SH UK

**Keywords:** Vaccination, Immunisation, Primary care, Organisational management, Systematic review

## Abstract

**Background:**

The vaccine system in England underwent radical changes in 2013 following the implementation of the Health and Social Care Act. There have since been multi-year decreases in coverage of many vaccines. Healthcare professionals have reported finding the new system fragmented and challenging. This study aims to produce a logic model of the new system and evaluate the available evidence for interventions to improve coverage.

**Methods:**

We undertook qualitative document analysis to develop the logic model using process evaluation methods. We performed a systematic review by searching 12 databases with a broad search strategy to identify interventions studied in England conducted between 2006 and 2016 and evaluated their effectiveness. We then compared the evidence base to the logic model.

**Results:**

We analysed 83 documents and developed a logic model describing the core inputs, processes, activities, outputs, outcomes and impacts of the new vaccination system alongside the programmatic assumptions for each stage. Of 9,615 unique articles, we screened 624 abstracts, 45 full-text articles, and included 16 studies: 8 randomised controlled trials and 8 quasi-experimental studies. Four studies suggest that modifications to the contracting and incentive systems can increase coverage, but changes to other programme inputs (e.g. human or capital resources) were not evaluated. Four multi-component intervention studies modified activities and outputs from within a GP practice to increase coverage, but were part of campaigns or projects. Thus, many potentially modifiable factors relating to routine programme implementation remain unexplored. Reminder/recall systems are under-studied in England; incentive payments to adolescents may be effective; and only two studies evaluated carer information.

**Conclusions:**

The evidence base for interventions to increase immunisation coverage in the new system in England are limited by a small number of studies and by significant risk of bias. Several areas important to primary care remain unexplored as targets for interventions, especially modification to organisational management.

**Electronic supplementary material:**

The online version of this article (10.1186/s12889-018-6228-5) contains supplementary material, which is available to authorized users.

## Background

The system for delivering vaccinations to the population in England underwent a radical change in 2013 following the implementation of the Health and Social Care Act 2012 [1]. Although General Practitioners (GPs) in primary care clinics (GP practices) retain responsibility for the delivery of vaccinations to their population, the organisations involved in agreeing the schedule of recommended vaccines, commissioning and funding of the service, and the collection and analysis of epidemiological data, changed radically with the formation of new organisations including NHS England (NHSE), Public Health England (PHE) and NHS Digital (NHSD). A tripartite group of organisations with responsibility for vaccination in England (the Department of Health (DH), PHE and NHSE) then went about developing and implementing a new operational model. This reorganisation was extremely disruptive to the delivery of the vaccination programme and the outcome has been described as *“fractured”* resulting in a *“complex mesh”*, causing difficulties for professionals delivering vaccinations on the ground [2]. At the same time the schedule of routine vaccines has continued to increase in size and complexity, now with 16 childhood vaccinations given to 6 age groups; 2 or 3 vaccines for adolescents; and 3 in older adults [3].

These factors may have influenced vaccine coverage. Although coverage for core childhood vaccinations remains high overall, downward trends have emerged since 2012 for several important vaccines, for example, measles, mumps and rubella vaccine (MMR) at 2 years decreased for the 3^rd^ consecutive year and currently stands at 91.6%, below the 95% target set by the World Health Organisation [4]. This is particularly concerning given the 5 recent outbreaks in England and the measles epidemic currently affecting many other European countries [5, 6]. Similarly, coverage of pentavalent vaccine (containing diphtheria, tetanus, pertussis, polio and haemophilus inflenzae b) by 12 months has decreased each year since 2012 [4]. Coverage is also not evenly distributed, with specific geographic areas having significantly lower coverage than the England average. This is particularly important in London where the lowest coverage is found for all childhood vaccines [4]. Similar trends are found in vaccine coverage in adults. For example, shingles vaccine was introduced into the schedule in 2015 for people aged over 70 years, with a catch-up campaign for people over 78. However, this year coverage is only 34.6% and 34.8% in each of these age groups respectively, which is 5.5% and 5.3% lower than the preceding year and shows a downward trend [7]. While demographic and socio-economic factors are well known to impact vaccine coverage, resulting in significant inequities between population groups, [4, 8–19] the contribution of health service organisation is unknown. GP Practices are independent contractors to the NHS and so organisational management data relating to their operating structure and function are not readily available. Although GP Practices are critical for successful delivery of the programme, within the post-2013 system there is also much less advice and support available from both NHSE and PHE, compared to the previous model, leaving practices isolated [2].

Many systematic reviews have been conducted evaluating interventions to improve coverage in high income countries: in specific population groups, including children, [20], adolescents, [21] people over 60, [22], children with high-risk conditions, [23] and looked-after children; [24] in specific scenarios, including reducing hesitancy, [25] missed opportunities, [26] and inequalities; [27] and evaluating specific types of interventions, including reminder/recall systems, [28, 29] mobile phone messages, [30] education, [31] eHealth, [32] primary care service delivery, [33] health worker reminders, [34] and financial incentives for doctors, [35] and patients [36]. However, there has been no consideration of how this evidence has been applied in the England context, nor whether the outcomes of any trials match those in the broader evidence. Therefore, the aims of this study are to i) develop and describe a logic model for the implementation of the vaccination programme in England; ii) undertake a systematic review of interventions that have modified vaccination programme delivery in England; iii) compare the evidence in England to existing evidence from high-income countries; and iv) evaluate how this evidence relates to critical components of the logic model to identify potential targets for improvement to increase coverage

## Methods

### Document analysis and logic model

**Fig. 1:**
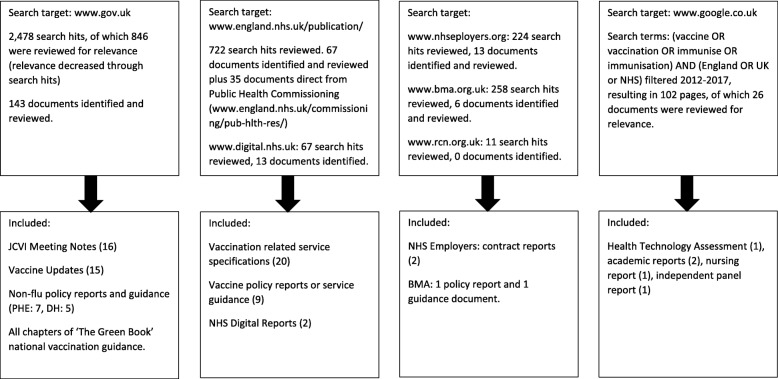
grey literature search strategy for document analysis

The purpose of developing the logic model is to accurately describe the components of the system for delivering the routine vaccination programme, including the underlying programmatic assumptions. To develop the model, we undertook a systematic analysis of documents published by organisations involved in designing and delivering the routine vaccination programme in England. We searched the websites of the following organisations for relevant documents: UK Government, including Department of Health and Public Health England (www.gov.uk), NHS England (www.england.nhs.uk), NHS Digital (www.digital.nhs.uk), British Medical Association (www.bma.org.uk), Royal College of Nursing (www.rcn.org.uk), and conducted a general search through Google (www.google.co.uk). Search terms consisted of “vaccination” and “immunisation” and spelling variants (Fig. 1). The focus was on documents relevant to the system since the implementation of the 2012 Health and Social Care Act, i.e. 2013-2017. We uploaded the included documents into NVIVO (v11) for qualitative analysis using document analysis methods [37].

The content of each included document was coded using deductive codes derived from the categories in logic model development (inputs, processes, activities, outputs, outcomes and impact). These were then synthesised and used to form a logic model with accompanying programmatic assumptions using the principles of process evaluation [38].

### Systematic review

We designed the systematic review to identify interventions that have been conducted within vaccine delivery programme in England. This has been reported in line with the PRISMA Guidelines for Systematic Reviews [39].

#### Search strategy

We searched Medline, ASSIA, Campbell Collaboration, Cochrane Database of Systematic Reviews, Embase, EPPI Centre, Psych Info, Web of Science, SCOPUS, Social Policy and Practice, Health Systems Evidence and Health Management Information Consortium using the search strategy shown in Additional file 1.

#### Inclusion and exclusion criteria

Studies with the following characteristics were included:

- Population: any in England
- Dates: published between 1996 and 2016
- Study design: Randomised Controlled Trial (RCT), quasi-experimental (including time-series and before-and-after studies) and ecological studies.
- Interventions: any designed to increase vaccine uptake or coverage, including any associated economic analyses.

Due to the complex and diffuse nature of the immunisation delivery system in England, we allowed for a wide range of study designs to be included. Additionally, we reviewed the references for other systematic reviews on similar topics that could have contained studies that fitted our inclusion criteria.

#### Study selection process

One reviewer screened articles by title and manually de-duplicated records (TCB). Two reviewers screened potentially relevant abstracts independently (TCB, SMJ). Any disagreement was resolved by discussion, based on the inclusion criteria. Both reviewers agreed the final inclusions.

#### Data extraction and assessment of bias

We extracted the following data from each included study for comparison: design, dates, population, intervention, vaccine(s), comparison, sampling method, allocation method, randomisation method, blinding, outcome measure and effect measure.

For RCTs, risk of bias was evaluated using the Cochrane Collaboration tool, with aspects of each study relating to selection bias, performance bias, detection bias, attrition bias, reporting bias and other bias extracted [40]. The Cochrane Collaboration tool is not specific to non-randomised study designs, and so, for quasi-experimental study designs, risk of bias was evaluated with the relevant Study Quality Assessment Tool published by the USA National Heart, Lung and Blood Institute, which are available for a variety of non-randomised study designs [41]. Both reviewers undertook assessment independently then compared outcomes and resolved differences by discussion.

## Results

### Logic model

For the grey literature search 2,230 search hits generated 303 documents that were reviewed for inclusion (Fig. 1). The components of the logic model were identified from 83 documents from the following sources:

- NHS England (NHSE): service specifications (*n*=20) and policy or guidance reports (*n*=9)
- Public Health England (PHE): Vaccine Updates published in 2017 (*n*=15), other policy or guidance reports (*n*=7), and The Green Book.
- Department of Health (DH): policy reports (*n*=5), Joint Committee on Vaccines and Immunisation (JCVI) meeting notes for 2013-2017 (*n*=16)
- NHS Employers: contract reports (*n*=2)
- NHS Digital (NHSD): reports (*n*=2)
- British Medical Association (BMA): reports or guidance (*n*=2)
- Other relevant academic or grey literature (*n*=5)

### Purpose

The purpose of the routine vaccination programme is described in the NHS Public Health Functions Agreement 2017-18 Core Service Specification National Immunisation Programme document, published by PHE and NHSE, as *“to protect the population from vaccine preventable diseases and reduce the associated morbidity and mortality.”* [42]. For the purposes of developing this logic model the intervention was defined as: the immunisation of individuals with pre-specified characteristics (e.g. age, co-morbidity, pregnancy) with immunological agents delivered by vaccination to prevent morbidity and mortality from vaccine preventable diseases (VPD) and reduce microbial spread within the population. The focus of this paper is the delivery of vaccinations through primary care facilities, so this is considered as the core activity.

### Logic model and assumptions

**Fig. 2:**
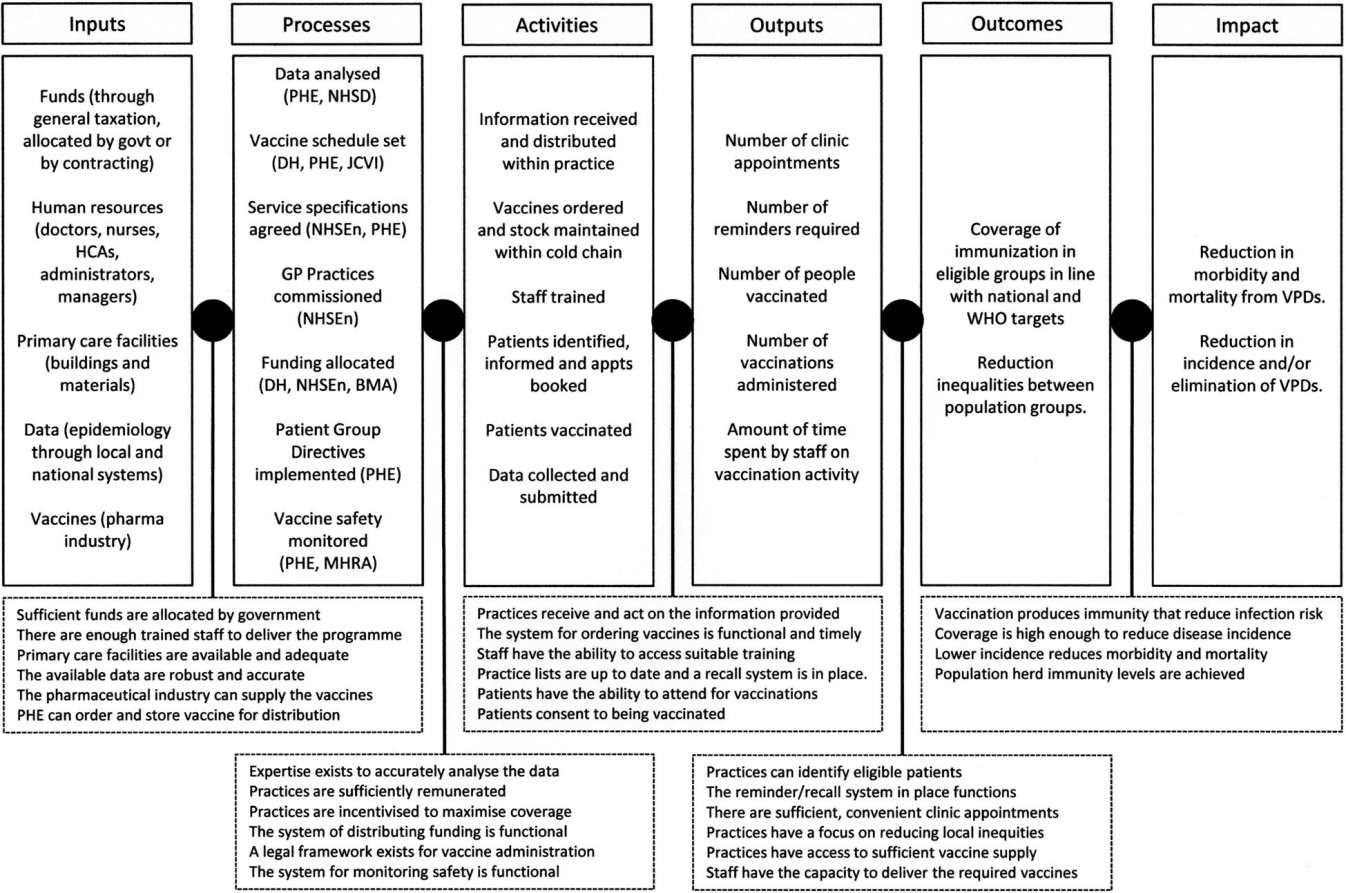
Logic model of the process of delivering the routine vaccine schedule in England with underlying assumptions

The logic model derived from the document analysis is presented in Fig. 2. Alongside GP Practices, there are 7 core organisations identified within the system: Department of Health (DH), the government department responsible for health; Public Health England (PHE), an executive agency of the DH with responsibility for vaccination; the Joint Committee on Vaccines and Immunisations (JCVI), an advisory group of independent experts for which PHE acts as secretariat; NHS England (NHSE), an executive non-departmental part of the DH responsible for commissioning (buying) vaccination services; NHS Digital (NHSD), which provides data systems and published some coverage statistics; the British Medical Association (BMA), the trade union and professional association of doctors in the UK, and the General Practitioners’ Committee (GPC), which is responsible for negotiating commissioning arrangements; the Medicines and Healthcare Products Regulatory Agency (MHRA), an executive agency of the DH with responsibility for safety of vaccines.

#### Inputs

We identified five key inputs for the vaccine programme: i) funding, ii) human resources, iii) primary care facilities, iv) data and v) vaccines. Funding is raised by general taxation and allocated by the Secretary of State from DH to NHSE via the Section 7A Agreement for use in commissioning public health services, including immunisation. The primary assumption is that this funding is sufficient to provide the programme. Vaccines are not included in this funding and are instead bought directly by DH and stored at PHE. Human resources are considered primarily at the GP Practice level, with assumptions that there are sufficient staff locally who have access to all the required facilities and resources. The expectations of the core and specific requirements for immunisation service are set out in NHSE’s Core Service Specification for the National Immunisation Programme, and each of the individual service specifications for each vaccine.

#### Processes

Seven core processes were described in the documents: i) data collection analysis, ii) setting the vaccine schedule, iii) producing the service specifications, iv) commissioning arrangements through contracting, v) allocation of funding, vi) production of patient group directives, and vii) monitoring vaccine safety.

Data are collected through two parallel systems: the Child Health Information System (CHIS); and ImmForm [43]. There are multiple, regional CHIS providers in England. It is a historical system that is commissioned independently by NHSE and collects data on core childhood immunisations [44]. ImmForm is a data collection, analysis and ordering platform commissioned by DH and PHE that mainly contains data on newer vaccines. Data analysis is primarily undertaken by PHE, with some stored and published by NHSD.

The recommended vaccine schedule is set by DH, following recommendations from the JCVI with specialist input from PHE. NHSE is tasked with commissioning vaccination services from GP Practices in England as per the Section 7A Agreement and does this through the immunisation service specifications and the more general contracting arrangements, such as the General Medical Services (GMS) and Personal Medical Services (PMS) contracts. The contents of these are negotiated annually with the GPC. PHE staff embedded within NHS England’s regional area teams provide oversight of the implementation of the programme. Nurses and Healthcare Assistants (HCAs) are legally enabled to vaccinate specific population groups without an individual prescription using a Patient Group Directive (PGD). Safety is monitored both by PHE and the MHRA through individual reporting using the Yellow Card notification system, [45] and population level studies.

#### Activities at GP Practices

GP Practices are expected to provide a minimum set of activities with the aim of *“offering immunisation to 100% of eligible individuals in accordance with… guidance from DH, NHSE and PHE”* [42]. These are set out in the service specifications and The Green Book, [46] which is the handbook of guidance relating to immunisation, published and updated by DH and PHE, with advice from JCVI. Broadly, the activities include: contacting and vaccinating eligible patients; keeping accurate records; training staff annually; providing an accessible service; providing information to patients; collecting and submitting data; involving users in service design; considering vulnerable and under-immunised groups; managing vaccine stock; and maintaining the cold chain. The key assumptions are that practices are sufficiently resourced and incentivised to undertake these activities.

#### Outputs

The outputs expected from the GP Practice are not clearly defined. The focus in the specifications is on providing data on the number of vaccinations administered to the eligible population, although there is also some consideration of availability and uptake of appointments and the use of a reminder and recall system. There is no consideration of staff time, capacity or cost. The main assumptions are that: practices have accurate lists and make sufficient appointments available; the reminder/recall system is in place and functional; the system for ordering vaccine stock and maintaining the cold chain is in place; and that patients attend and consent to vaccination.

#### Outcomes

The outcomes are more clearly defined, with coverage of immunisation within eligible populations is a core focus of all the documents. National level coverage expectations are described in the Core Service Specification, through which DH holds NHSE to account, which are based on coverage achieved in previous years or global recommendations from the WHO. As a result, coverage levels are reported by PHE and considered by JCVI. There is also a statutory requirement to reduce inequalities between groups, specified as people with protected characteristics as defined in the Equality Act 2010 [47]. The assumptions here are that the systems and activities undertaken by the GP Practice’s work sufficiently well to vaccinate high levels of the local population and that these local data aggregate to these thresholds nationally.

#### Impacts

The purpose of the programme as stated in the Core Service Specification *“is to enable [NHSE] to commission national immunisation services to a standard that will continue to minimise the infections and outbreaks caused by vaccine preventable diseases… and... to protect the population from vaccine preventable diseases and reduce the associated morbidity and mortality.”* This aim is reflected as a disease specific aim in each of the individual service specifications. Much of the focus on vaccination policy at the JCVI is on reduction of circulating disease prevalence. Disease elimination is also a stated aim of the programme, for example, the Section 7A agreement contains the WHO European regional target to eliminate measles and rubella infections by 2020. The assumptions here are primarily that the vaccinations recommended in the schedule produce sufficient immunity to reduce disease incidence; and that coverage is high enough to reduce pathogen circulation to reduce outbreaks and move towards elimination.

### Systematic review

**Fig. 3:**
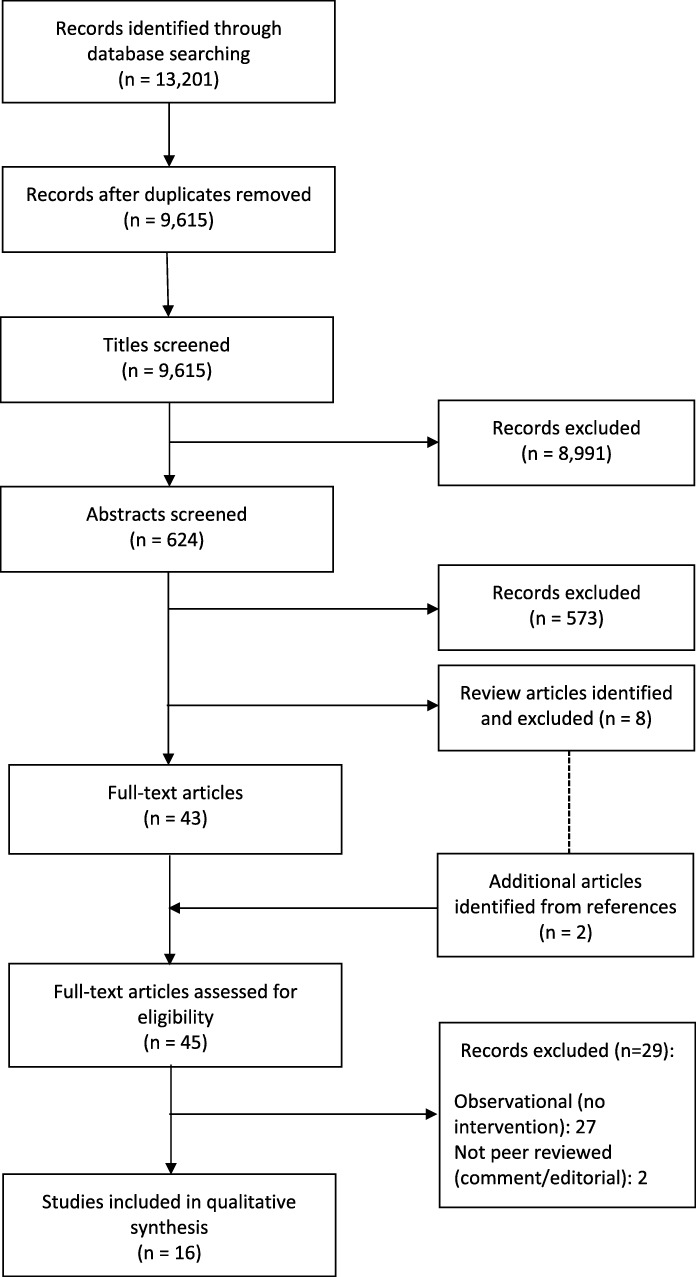
PRISMA flowchart of literature selection

The PRISMA Flow Chart of study selection and inclusion is presented in Fig. 3. Of 9,615 unique articles, 624 abstracts were screened, leading to 43 full text articles being reviewed. A further 2 studies were identified from the references of 8 other review articles [27, 33, 35, 36, 48–51].

In total 16 experimental studies were included: 8 RCTs (Table 1) and 8 quasi-experimental studies (Table 2). Four studies considered seasonal influenza, [52–55] with 2 more looking at influenza and pneumococcal vaccines together; [56, 57] 5 considered measles, mumps and rubella (MMR); [58–62] 3 the whole childhood schedule; [63–65] 1 each considered pneumococcal vaccines alone and human papillomavirus (HPV) vaccines; [66, 67] and 3 looked at the effect of the Quality Outcomes Framework (QOF), an incentive scheme, focusing on influenza coverage in specific risk groups [8, 68, 69].

**Table 1:** Characteristics and outcome of included randomized controlled trials

First Author, year [reference]	Sample from population	Vaccine	Intervention category	Intervention vs. comparison	Total sample (intervention/comparison)	Effect measure	Risk of bias
Arthur, 2002 [52]	Patients aged over 75 from a large rural general practice in Leicestershire.	Influenza	Reminder/recall & outreach	Personal letter of invitation to attend for vaccination vs. health check at home where vaccine was offered.	2052 (1372/680)	OR 1.28 (CI 1.03-1.58).	High risk of performance bias (no blinding) and attrition bias (high decline of health checks).
Hull, 2002 [53]	Patients aged 65-74 from at 3 general practices in London & Essex.	Influenza	Reminder/recall	Telephone call from receptionist to make appointment vs. letter & info leaflet alone.	1318 (660/658)	Adj OR 1.29 (CI 1.03-1.62) for phone call (*p*=0.026)	Low risk of bias.
Nuttall, 2003 [54]	Previous non-attenders aged 65-90 from a general practice in Lancashire.	Influenza	Reminder/recall & outreach	Letter vs. leaflet vs. letter & home visit to discuss vaccination.	90 (30/30/30)	OR 0.84 (0.26-2.74; *p*=0.77) [50]	Generally low, but risk of performance bias (no blinding)
Herrett, 2016 [55]	Patients aged 18-64 in risk groups from 153 general practices in London.	Influenza	Reminder/recall	Sending of pre-defined, recommended text message reminders vs. usual care.	102257 (77 practices/ 79 practices)	OR 1.12 (CI 1.00-1.25)	Low risk of bias.
Porter-Jones, 2009 [58]	All children eligible for 1^st^ MMR from Flintshire, Wales.	MMR	Patient education	Teddy bear with t-shirt directing parents to information sources vs. no bear	974 (542/432)	OR 1.06 (CI 0.73-1.57)	High risk of selection bias (convenience sampling, no allocation concealment) and performance bias (lack of blinding).
^a^Shourie, 2013 & Tubeuf 2014 [59, 74]	First-time parents with a child aged 3-12 months eligible for MMR from 512 general practices in the North of England.	MMR	Patient education	Web-based decision aid vs. leaflet & usual practice vs. usual practice alone.	220 (48/85/70)	Non-significant difference due to small sample: leaflet vs. usual practice OR 0.14 (CI 0.02-1.14);); and decision aid vs. leaflet OR 10.6 (CI 0.1-188.5); decision aid vs usual practice 2.1 (CI 0.1-52.5)	Generally low, however small groups and lack of blinding.
Siriwadena, 2002 [56]	Patients aged >65 and those in eligible risk groups from 30 general practices in Trent region.	Influenza & pneumococcal	Healthworker education	Educational visit to GP practices based on principles of academic detailing lasting one hour vs. provision of information on performance alone.	30 practices (15/15)	Increases in uptake of pneumococcal in patients with CVD (1.23, CI 1.13-1.34; *p*=<0.001) and diabetes (1.18, CI 1.08-1.28; *p*<0.001). No difference in any group for influenza	Generally low, but unclear risk of selection bias (randomisation not described) and high risk of performance bias (analysts not blinded)
Mantzari, 2015 [67]	Females aged 16-18 eligible for HPV vaccine in Birmingham and East North health region.	HPV vaccine	Incentive & reminder/recall	Vouchers worth £45 for completing vaccine course of 3 injections and text message reminders vs. invitation letter alone.	1000 (500/500)	First dose: OR 1.63 (CI 1.08-2.47) for first time and 1.63 (1.075-2.472) for previous non-attenders. Third dose: OR 2.15 (CI 1.32-3.96) for first time and 4.28 (CI 1.92-9.55) for previous non-attenders.	Unclear risk of detection bias (possible for analysis to identify groups) otherwise low.

*MMR* measles, mumps and rubella vaccine, *HPV* human papillomavirus, *CI* 95% confidence interval, *Adj* adjusted, *OR* odds ratio, *CVD* cardiovascular disease

^a^both studies report analysis and results from the same sample

**Table 2:** Characteristics and outcome of included quasi-experimental studies

First Author, year [reference]	Sample from population	Design	Vaccine	Intervention category	Intervention	Sample and comparison	Effect measure	Risk of bias
Le Menach, 2014 [60]	Children aged 6 months – 16 years from one general practice.	B&A	MMR	Multi-component	Campaign offering accelerated vaccination (6-11 months), early 2nd dose (6-11 months) and catch up vaccinations.	Coverage in 1538 children measured before and after campaign.	Increase in proportion of >14 months immunised by 3% (to 71%) and of >60 months by 5% (to 65%) following the campaign.	Low (assessors not blinded; unclear consideration of group effects)
Cockman, 2011 [61]	All children in the London Borough of Tower Hamlets.	Eco	MMR	Multi-component	Quality improvement project associated with campaign including: incentive payments; practice network; commissioning care package; new targets; IT for reminder/recall; active follow-up defaulters.	Coverage in all children in the area measured over time.	Coverage of MMR1 increased from 80% before the intervention to 94% after. Significant difference (*p*=0.001) in slope of coverage change pre (-0.07% per quarter) and post (1.86% per quarter).	Low (assessors not blinded; unclear consideration of group effects)
Siriwardena, 2003 [57]	Selected general practices from all practices in Lincolnshire.	B&A	Influenza & pneumococcal	Multi-component	Dissemination of clinical guidelines; advise on data and surveillance; organisational strategy; reminder/recall; comparative performance.	Coverage in 21 general practices before and after participating in project.	Significant increases in coverage before and after for both influenza and pneumococcal in a range of groups e.g. pneumococcal in CHD 27.5% increase (CI 12.6-42.3%; *p*=0.002)	Moderate (general objective; diffuse intervention; assessors not blinded; no consideration of group effects)
McDonald, 1997 [66]	Eligible patients in risk groups registered at general practices in Tameside.	B&A	Pneumococcal	Multi-component	Improved vaccine supply; clinical guidelines; patient materials; patient information leaflet translations; education.	Proportion of eligible patients immunised at participating practices before and after the intervention.	Increase in coverage from 6% before to 33% after the campaign.	Moderate (eligibility and selection unclear; diffuse intervention; assessors not blinded; no consideration of group effects)
MacDonald, 2016 [62]	Unimmunised children from Dudley local health area.	Eco ITS	MMR	Outreach	Immunisation offered during home visits	Comparison of coverage in local population using quarterly routine data.	Intervention contributed 2.6% of the MMR doses given during the study period.	High (enrolment and sample size unclear; assessors not blinded; limited statistical consideration)
Atchison, 2013 [63]	General practices in Wandsworth, London.	B&A	Childhood schedule	Reminder/recall	Standardised reminder/recall system involving letters and referral to health visitors.	32 participating practices compared to 44 not participating before and after the intervention.	Significant increase in coverage in intervention group, but as a result of unexplained decreases in control group coverage.	Moderate (likely differences between intervention and control practices; assessors not blinded; no consideration of group effects).
Henderson, 2004 [64]	General practices in Highland NHS Health Board area.	Eco	Childhood schedule	Reminder/ recall	Participation in national reminder/recall system vs. use of general practices’ own system.	Coverage between 8 practices using their own reminder/recall system vs. 66 participating in a national system.	Higher coverage in national system practices of diphtheria by age 2 (6.4%, CI 1.7-11.1, *p*=0.001) and Men C by age 2 (7.6%, 2.7-13%, p=0.001) but no difference for diphtheria by 1 year or MMR by 2 years.	Moderate (natural experiment – intervention dose unclear; likely difference between intervention and control practices; assessors not blinded; group effects not considered)
Gosden, 2003 [65]	Selected general practices in England.	B&A	Childhood schedule	Incentive	General practice contracting arrangements: GMS vs. PMS contracts.	Coverage in 10 practices who had switched to PMS contract vs. matched 10 control practices on GMS contract.	No difference in immunisation coverage between practices (-1.08%, CI -17.95-15.8%)	Moderate (natural experiment – intervention dose unclear; likely difference between intervention and control practices; unclear if large enough sample; assessors not blinded; group effects not considered)
Norbury, 2011 [8]	315 general practices in Scotland	Eco B&A	Influenza in >65-year olds and risk groups	Incentive	QOF	Coverage in >65 yo and risk groups before (03-04) vs. after (06-07) introduction on QOF incentive in 2004.	Increase in coverage by 3.5% (CI 3.3 to 3.7%); higher in <65 yo 8.8% (CI 8.3 to 9.4%) than >65 yo 3.3% (CI 3.1 to 3.6%). Higher increases in those with disease risk, than age alone.	Low (no blinding of assessors)
Kontopantelis, 2012 [68]	All practices in England.	Eco CB&A	Influenza in people with CHD	Incentive	QOF	Coverage before vs. after the increase in upper payment threshold from 85% to 90% in 2006; and vs. other risk groups with no threshold change.	Immediate increase of 0.41% (CI 0.25 – 0.56%) population coverage, with larger increase seen in practices with <85% in 2006 of 0.85% (0.62 – 10.08%)	Low (no blinding of assessors)
Kontopantelis, 2014 [69]	Patients at 50 representative practices from 644 in CPRD	Eco ITS	Influenza in people with asthma	Incentive	QOF	Coverage before vs. after QOF target removed in 2006.	Small drop in coverage -0.70% (CI -1.1% to -0.39%	Low (no blinding of assessors)

*B&A* before and after study, *ITS* interrupted time series, *Eco* ecological, *MMR* measles, mumps and rubella vaccine, *PMS* personal medical services contract, *GMS* general medical services contract, *QOF* Quality Outcomes Framework, *CI* 95% confidence interval, *OR* odds ratio

Due to heterogeneity in study populations and intervention types meta-analysis was not possible, except in one case where it had already been performed elsewhere in the literature.

### Randomised controlled trial interventions

Four studies examined reminder/recall interventions in increasing uptake of seasonal influenza vaccination in adults [52–55]. Two of these combined reminders to eligible patients with a home visit component, compared to reminders alone [52, 54]. One study had a high risk of performance and attrition bias and the other had a low risk of bias but was under-powered (90 subjects). In a previous systematic review and meta-analysis evaluating interventions to increase uptake of influenza vaccine in people over 60, the pooled effect of these studies involving 710 intervention patients (who received a reminder and a home visit) and 1402 controls with ‘usual care’ was an odds ratio (OR) of 1.30 (95% confidence interval (CI) 1.05-1.61, p=0.01). However, neither evaluated the additional cost of the home visit component. Of the studies considering reminders alone, one compared a phone call from a receptionist to sending a letter and found an OR of 1.29 (CI 1.03-1.62) and the other evaluated using text message reminders at practice level, finding a non-significant OR 1.12 (CI 1.00-1.25) increase in uptake. Both provided evidence with low risk of bias.

Two studies used educational interventions to increase uptake of MMR vaccine. One used a teddy-bear with signposting to government information and found no difference between intervention and control groups and was at high risk of bias [58]. The other evaluated a web-based decision aid as compared to a leaflet and compared to usual practice alone and found OR 0.14 (CI 0.02-1.14) lower coverage in the leaflet group compared to usual practice (OR 0.14 (CI 0.02-1.14) ) and higher coverage in the decision aid group compared to the leaflet (OR 10.6 (CI 0.1-188.5)), but no difference between decision aid and usual practice groups [59]. However, sample size was small (220 in three groups) and confidence intervals were wide. An associated cost-effectiveness analysis found the decision aid was lower cost to deliver than usual practice (-£9.20) and leaflets (-£7.17), however no direct cost was assigned to the decision aid (e.g. development and maintenance costs).

One study focussed education on healthcare workers through use of an education visit to increase uptake of both influenza and pneumococcal vaccine in people aged over 65 years in specific risk groups at practice level [56]. It found mixed results with increases in coverage in some groups (e.g. OR 1.23, CI 1.13-1.34, pneumococcal uptake in patients with cardiovascular disease (CVD)) but not in others, making overall effect difficult to establish.

HPV vaccine is provided to adolescent females and a study at low risk of bias and a large sample (1,000 subjects), that compared the provision of a £45 voucher and text message reminders to an invitation letter alone found a significant increase in uptake of both first dose (OR 1.63, CI 1.08-2.47) and course completion (OR 2.15, CI 1.32-3.96) in the intervention group [67]. No evaluation of cost effectiveness was made.

### Quasi-experimental interventions

Most of the quasi-experimental studies use routine or population level data to evaluate the effects of either specific interventions to changes to vaccination programme implementation (Table 2). All included studies focus on changes in proportion of the eligible population covered over time, or differences in coverage between groups. Two studies with low risk of bias evaluated complex, multi-component campaigns to improve MMR uptake in a local population, although no evaluation of cost [60, 61]. One other study evaluated offering MMR vaccine during home visits, but was at significant risk of bias [62].

Two studies evaluated complex interventions to improve influenza and/or pneumococcal coverage in adults in risk groups. One that used quality improvement methods found significant increases in coverage across a range of vaccines, [57] and the other found an increase in coverage from 6% to 33%, so from a low start to a relatively low overall coverage level [66]. Both of these had a significant risk of bias.

A further two studies evaluated the effectiveness of reminder/recall interventions to increase coverage of all childhood vaccines in the schedule. One implemented a standardised, complex system of reminder letters and referral to health visitors in a London borough [63]. Although a significant increase in coverage was identified, this was due to an unexplained reduction in coverage in the control group, making the results difficult to interpret. The other compared practices in Scotland using a national reminder/recall system to those using their own local systems and found higher levels in coverage in the former group, but not across all vaccines [64].

Four studies evaluated the effects of various changes to the system of contracting and payments to GP Practices in England. The first evaluated differences between practices contracted using the Personal Medical Services (PMS) contract compared to the General Medical Services (GMS) contract. The ability to switch from the standard GMS to the new PMS contract occurred in 1999 and a relatively small number of practices did so. Under the GMS contract, GPs were incentivised to increase practice list size and meet targets for providing specific services (such as immunisation) to maximise income. The PMS contract was designed to be more locally responsive with lower levels of administration and with the GPs in receipt of a salary. Overall the study found no differences between GMS and PMS practices over time, although only 10 PMS practices were included [65]. Another change to the arrangements to pay GPs was the introduction of the Quality Outcomes Framework (QOF) in 2004, a pay for performance system that incentivised GPs to meet specific quality targets [70]. This included several targets for influenza coverage in specified risk groups, including patients with asthma, diabetes and heart disease, among others. One study evaluated the effect of the introduction of these targets in Scotland and found an overall increase in coverage in 3.5% (CI 3.3-3.7%), but that this was far greater in incentivised populations (aged under 65 in risk groups: 8.8%, CI 8.3-9.4%) that in the population eligible by age alone (>65 years: 3.3%, CI 3.1-3.6%). Another study evaluated the effect of raising the threshold for payment from 85% to 90% for influenza coverage in people with cardiovascular disease in 2006 across all practices in England and found a small increase overall (0.41%, CI 0.25 – 0.56%), but a larger increase seen in practices with <85% coverage in 2006 of 0.85% (0.62 – 10.08%) [68]. In 2006 the QOF target and payment for influenza coverage in people with asthma was removed and a study was conducted in 644 practices signed up to the Clinical Practice Research Datalink (CPRD) to evaluate the overall effect, which found a small drop in coverage of -0.70% (CI -1.1% to -0.39%) [69]. Together these studies provide evidence with low risk of bias that changes to the contracting and incentive payment system can affect coverage levels, but suggest that the overall effect is likely to be small.

## Discussion

In this this paper we have presented a logic model of the theoretical structure of the vaccination programme in England following the implementation of the HSCA in 2013 and identified and described the underlying assumptions that allow it to function. This will enable further work evaluating the fidelity of the implementation of this system at GP Practice level, which has not yet been studied. Through the systematic review, we have identified which aspects of the system have been modified to improve coverage, and will now compare the evidence from England to the available evidence from other high-income countries. Overall, we identified a relatively small number of experimental studies (*n*=16). All studies used uptake or coverage to be the outcome of interest, rather than disease incidence or another impact indicator. Few of the interventions evaluated modification to routine activities and outputs within a GP Practice. Of those that did make changes at GP Practice level the modifications were associated with specific time-limited projects, [57, 63] or campaigns [60, 61]. Thus, potentially modifiable factors relating to programme implementation at GP Practice level remain unexplored. Using the logic model shown in Fig. 2 we have compared this evidence to areas of programme implementation to identify un-researched areas and potential targets for future studies, and to draw lessons for policy-makers.

### Inputs and processes

Four studies that modified the contracting and payment systems for GP Practices in England increased coverage. This requires modification of both the inputs (financing) and processes (contracting). A Cochrane Review originally conducted in 2000 and updated in 2009 did not find enough evidence to support the use of target payments to increase vaccine coverage, [48] and another Cochrane Review published in 2011 considering incentives for primary care physicians more broadly concluded that there is growing evidence for their use in improving quality of care, but the evidence remains limited [35]. This is likely in part due to the restriction of the evidence to RCTs, as the evidence identified here was from quasi-experimental studies using ecological data. The four studies that evaluated the impact of the introduction and changes to the QOF incentive scheme for GP Practices found that the availability of incentives is likely to increase coverage, but only by a small amount. Currently, only influenza vaccine in people with risk factors are incentivised this way and there may be merit in considering adding other vaccines to the QOF system, if coverage is low. No other incentive or financing schemes were evaluated, and this remains a significant gap in the evidence base, and there is the potential for NHSE and PHE to develop and trial other forms of incentive schemes. Additionally, other process elements, such as modifying the legal framework for delivering vaccines (e.g. HCAs to deliver more vaccines); or non-financial programme inputs (e.g. provision of additional facilities) have not been considered.

### Activities and outputs

Most of the studied focussed on specific activities that often were developed or existed outside of the GP practice structure, including reminder/recall systems (*n*=6), campaigns (*n*=2), or involving elements of outreach (*n*=3). Only four modified elements within the GP practice itself. One provided health worker education and 3 of the multi-component interventions modified a wide variety of elements of service delivery. These provide some evidence for effectiveness, although in some cases had significant risk of bias. There is wider international evidence that multi-component interventions can be effective at reducing inequalities in immunisation coverage in deprived, urban areas [27]. However, despite the chronic problems with low coverage in London, few well conducted intervention studies were identified [12]. Of those that were identified, the interventions were vaccine specific campaigns, [60, 66] which may not be easily reproducible, or highly diffuse interventions modifying many aspects of routine service delivery, [57, 61], which may not be relevant to other contexts. Overall, there was little consideration of the effects of interventions on core programme outputs, such as staffing levels, task shifting, information provision, service delivery models, or cost structures within GP practices. Organisational factors associated with immunisation coverage at GP Practice level have been widely studied using cross-sectional surveys. For example, a survey involving 759 practices in England evaluated factors associated with high levels of influenza coverage and found the following to have a significant independent association: identified lead staff member; written report of practice performance; personal invitation to patients; aiming for QOF targets; and using IT systems to identify patients [71]. A similar survey was conducted with 257 GP Practices in a region of England to identify factors associated with MMR coverage [72]. It found no association with practice size or number of staff (GPs or nurses); however having a strategic approach to MMR coverage and identifying clear objectives (e.g. target >90%) were associated with higher coverage. Modifications to the organisation of immunisation services at GP Practices should be a topic of further research and look promising as a target to increase coverage.

Robust and reliable reminder/recall systems are a core component of any vaccine programme and have very good, reproducible evidence for effectiveness [29]. Despite the risk of bias in several of the studies identified in this review, there is some evidence of effectiveness in the England context specifically, although more research would be required to identify the most cost-effective method.

Only one study considered the role of Health Visitors (HVs – community public health nurses) as part of an outreach campaign to increase MMR coverage in one area of the UK, [62] but this was compromised by very high risk of bias. One of the RCTs that had a high risk of bias found that home visits could increase coverage of influenza vaccine in older people, [73] and this may warrant repeating in areas of low coverage, with consideration of cost-effectiveness. Outreach work to improve child health, including immunisation, is usually conducted by HVs, who had formerly been based in GP Practices. In 2015, however, commissioning of HV services was moved to Local Authorities and most HVs left GP Practices to other locations. Currently the primary focus of a HV is to improve outcomes in 6 ‘high-impact areas’, which do not include immunisation. A Health Technology Assessment review of the impact of HVs on child health outcomes, published in 2000, found good evidence for effectiveness [51]. This was not supported by the evidence identified in this study, possibly due to the nature of multiple restructures of HV services since 2000.

Surprisingly, only two RCTs studied the effect of information provided to patients or carers, and both were designed to improve uptake of MMR vaccine, due to the historical controversy in this area. The provision of signposting information on a teddy-bear was not found to be an effective method, although the study was at high risk of bias, [58] and a web-based decision aid produced mixed results with small intervention groups, making firm conclusions difficult to draw [59]. None of the other intervention studies considered reducing vaccine hesitancy as an explicit aim, which is in line with the wider available evidence from other high-income countries [25]. Improving the information provided to patients would be a key area for future evaluation in the England context.

One study found that provision of a financial incentives with reminder/recall messages to adolescent females could increase uptake of HPV vaccine [67]. An extensive Health Technology Assessment evaluating incentives for parents of children eligible for vaccination, published in 2015, found limited evidence of effectiveness overall, but concluded that incentives for parents might not be acceptable, but if introduced they should be universal and not targeted [36]. If coverage in adolescent vaccines is low, then incentives could be further explored in this population.

### Outcomes and impact

Evidence of effectiveness of all included studies was measured as changes in vaccine coverage levels. None considered overall impacts such as reduction in cases of VPDs, or overall morbidity and mortality. However, this is likely to be considered on a national level by PHE independent of the research evidence. Although reduction in inequalities is a key outcome of the programme, only one study considered the reduction in inequalities specifically [61]. Focusing vaccine campaigns or outreach interventions in areas of higher disease incidence or outbreaks may enhance the effectiveness of such programmes and provide useful evidence if evaluated.

### Lessons for policy

When compared to the logic model, we identified several areas where interventions are available to support modifications to the existing system to improve coverage. These include: multi-component interventions that improve service quality in geographic areas of low coverage; incentive payments to adolescents; effective reminder/recall systems; potential use of outreach programmes; and possible modifications to contracting and incentive payments. There are also several areas of programme implementation that have not been evaluated and could be potential future targets for policy changes or interventions, including: task shifting; additional non-financial resource inputs; information to patients; health worker training; and changes to organisational management within a GP practice.

### Limitations

The limitations of this study include: the biased nature of the available evidence; diffuse nature of the interventions; the small number of studies overall; and the limited number of studies for different categories of interventions. Thus, the conclusions drawn here should be approached with caution.

## Conclusions

The process of delivering the routine immunisation programme through GP Practices in England is well described, but contained across a wide range of documents. This has been synthesised into a clear logic model with underlying assumptions that will be valuable to policy-makers and researchers to develop and test interventions in the context of declining national immunisation coverage. The evidence base for interventions to increase immunisation coverage in the England context are limited by a small number of studies for different categories of interventions; and by significant risks of bias in much of the evidence base. Several areas remain unexplored as targets for interventions, especially modifications to the organisational management of GP Practices.

## Additional file

Additional file 1: Medline Search Strategy. (DOCX 15 kb)

## Abbreviations

B&A: Before and after study design
BMA: British Medical Association
CB&A: Controlled before and after study design
CVD: Cardiovascular disease
CHIS: Child Health Information System
CI: 95% confidence interval
DH: Department of Health
GMS: General Medical Service contract
GP: General Practitioner
GPC: General Practitioners Committee
HCA: Healthcare Assistant
HPV: Human papillomavirus
HSCA: Health and Social Care Act
HV: Health visitor
ITS: Interrupted time series study design
JCVI: Joint Committee on Vaccines and Immunisations
MHRA: Medicines and Healthcare Products Regulatory Agency
MMR: Measles, mumps and rubella vaccine
NHSD: National Health Service Digital
NHSEm: National Health Service Employers
NHSE: National Health Service England
OR: Odds ratio
PHE: Public Health England
PMS: Personal Medical Services contract
QOF: Quality and Outcomes Framework
RCT: Randomised controlled trial
VPD: Vaccine preventable disease
WHO: World Health Organisation
