# Supplementary material for: Vaccination in England: a review of why business as usual is not enough to maintain coverage
Source: BMC Public Health. 2018 Dec 6;18:1351. doi: 10.1186/s12889-018-6228-5 (PMC6282278; doi:10.1186/s12889-018-6228-5)
Supplement: Supplementary file 1 — Medline Search Strategy. (DOCX 15 kb) [file 12889_2018_6228_MOESM1_ESM.docx]

**Additional file 1: Medline Search Strategy**

1 exp Vaccination/

2 exp immunization/

3 exp immunization programs/

4 (immuni*ation or immuni*e or vacci*).mp. [mp=title, abstract, original title, name of substance word, subject heading word, keyword heading word, protocol supplementary concept word, rare disease supplementary concept word, unique identifier, synonyms]

5 ((vacci* or immuni*) adj3 (program or programme or schedule)).mp. [mp=title, abstract, original title, name of substance word, subject heading word, keyword heading word, protocol supplementary concept word, rare disease supplementary concept word, unique identifier, synonyms]

6 1 or 2 or 3 or 4 or 5

7 (uptake or cover* or impact* or cover* or receipt or accept* or complete* or prevalence or up to date).mp. [mp=title, abstract, original title, name of substance word, subject heading word, keyword heading word, protocol supplementary concept word, rare disease supplementary concept word, unique identifier, synonyms]

8 (delivery or clinic* or appointment* or staff or doctor* or nurse* or train* or fund* or incentive* or payment or commission* or organi* or phone* or text* or message* or online or remind* or letter or post or mail or outreach or home visit* or money or cash or invit* or time or protocol or standing order* or marketing or media).mp. [mp=title, abstract, original title, name of substance word, subject heading word, keyword heading word, protocol supplementary concept word, rare disease supplementary concept word, unique identifier, synonyms]

9 (delivery or system or policy or governance or management or manager* or organi*ation or evidence or context or culture or factor* or variable*).mp. [mp=title, abstract, original title, name of substance word, subject heading word, keyword heading word, protocol supplementary concept word, rare disease supplementary concept word, unique identifier, synonyms]

10 exp Health Services/

11 exp Health Services Accessibility/

12 exp "Delivery of Health Care"/ or exp "Delivery of Health Care, Integrated"/

13 7 or 8 or 9 or 10 or 11 or 12

14 exp Family Practice/ or exp General Practice/

15 exp Primary Health Care/

16 (communit* or primary care or communit* care or family medicine or general practice or family practice or local health service*).mp. [mp=title, abstract, original title, name of substance word, subject heading word, keyword heading word, protocol supplementary concept word, rare disease supplementary concept word, unique identifier, synonyms]

17 14 or 15 or 16

18 6 and 13 and 17

19 exp united kingdom/

20 (united kingdom or UK or great britain or britain or british isles or england or wales or scotland or northern ireland).mp. [mp=title, abstract, original title, name of substance word, subject heading word, keyword heading word, protocol supplementary concept word, rare disease supplementary concept word, unique identifier, synonyms]

21 19 or 20

22 18 and 21
